# PAX5-miR-142 feedback loop promotes breast cancer proliferation by regulating DNMT1 and ZEB1

**DOI:** 10.1186/s10020-023-00681-y

**Published:** 2023-07-04

**Authors:** Zhao-Hui Chen, Yi-Bo Chen, Hao-Ran Yue, Xue-Jie Zhou, Hai-Yan Ma, Xin Wang, Xu-Chen Cao, Yue Yu

**Affiliations:** 1 The First Department of Breast Cancer, Tianjin Medical University Cancer Institute and Hospital, National Clinical Research Center for Cancer, Huan-Hu-Xi Road, He-Xi District, Tianjin, 300060 China; 2 Key Laboratory of Breast Cancer Prevention and Therapy, Tianjin Medical University, Ministry of Education, Tianjin, 300060 China; 3 Key Laboratory of Cancer Prevention and Therapy, Tianjin, 300060 China; 4 Tianjin’s Clinical Research Center for Cancer, Tianjin, 300060 China; 5 Department of General Surgery, The Affiliated Hospital of Inner Mongolia Medical University, Hohhot, 010050 Inner Mongolia China

**Keywords:** miR-142-5p, miR-142-3p, PAX5, DNMT1, ZEB1, Breast cancer, Proliferation

## Abstract

**Background:**

Breast cancer is one of the most common malignancies occurred in female around the globe. Recent studies have revealed the crucial characters of miRNA and genes, as well as the essential roles of epigenetic regulation in breast cancer initiation and progression. In our previous study, miR-142-3p was identified as a tumor suppressor and led to G2/M arrest through targeting CDC25C. However, the specific mechanism is still uncertain.

**Methods:**

We identified PAX5 as the upstream regulator of miR-142-5p/3p through ALGGEN website and verified by series of assays in vitro and in vivo. The expression of PAX5 in breast cancer was detected by qRT-PCR and western blot. Besides, bioinformatics analysis and BSP sequencing were performed to analyze the methylation of PAX5 promoter region. Finally, the binding sites of miR-142 on DNMT1 and ZEB1 were predicted by JASPAR, and proved by luciferase reporter assay, ChIP analysis and co-IP.

**Results:**

PAX5 functioned as a tumor suppressor by positive regulation of miR-142-5p/3p both in vitro and in vivo. The expression of PAX5 was regulated by the methylation of its promoter region induced by DNMT1 and ZEB1. In addition, miR-142-5p/3p could regulate the expression of DNMT1 and ZEB1 through binding with their 3’UTR region, respectively.

**Conclusion:**

In summary, PAX5-miR-142-DNMT1/ZEB1 constructed a negative feedback loop to regulate the progression of breast cancer, which provided emerging strategies for breast cancer therapy.

**Supplementary Information:**

The online version contains supplementary material available at 10.1186/s10020-023-00681-y.

## Introduction

Breast cancer occurs commonly for female worldwide and is the leading cause of female tumor death for decades (Harbeck and Gnant 2017). As the breast cancer therapy improved continuously these years, the outcomes of breast cancer patients have been surprisingly developed (Harbeck and Gnant 2017; Fahad 2019). Nevertheless, certain subtypes of breast cancer patients are still suffering from disease and have unsatisfied prognosis (Tray et al. 2019; Li et al. 2017). Thus, emerging biomarkers or treatment targets need further investigation.

Recently, accumulating studies have focused on the dysregulation of cancer related genes and miRNAs (Rupaimoole and Slack 2017; Liang et al. 2020). microRNA (miR) is a class of non-coding RNAs with approximately 20–24 nucleotides and regulated gene expressions by binding to the 3′-untranslated regions (UTRs) of their target mRNAs (Lee and Dutta 2009). The miRNAs are generated starting from the transcription of long primary miRNAs (pri‐miRNAs) in nuclear, then are exported to cytoplasm for further processing and finally form the mature miRNA (Alarcon et al. 2015), which is initially a duplex, including a 3′ end originated 3p strand and a 5′ end derived 5p strand from the pre‐miRNA hairpin (Lu and Rothenberg 2018). Accumulating evidence has demonstrated the critical roles of miRNAs in multiple cancers, the pattern of which expression could be associated with cancer type, stage, and other clinical variables (He et al. 2020; Leva et al. 2014). Besides, miRNAs have been identified to act both as oncogenes and as tumor suppressors. For example, miR-133 is down-regulated in gastric cancer patients and negatively correlated with invasion depth, tumor size, and peripheral organ metastasis (Cheng et al. 2014). Other miRNAs such as miR-296 and miR-130a are also associated with tumor angiogenesis through interaction with pro-angiogenic receptors and antiangiogenic factors such as HGS, HOXA5, and GAX, respectively (Wurdinger et al. 2008; Chen and Gorski 2008). While in breast cancer, miR-182-5p was reported as a tumor promoter and involved in breast cancer progression by targeting CMTM7 in our previous study (Chen et al. 2023).miR-142-5p and miR-142-3p are two single-stranded miRNAs derived from the same RNA duplex and were first reported by Wu et al. in 2007 (Wu et al. 2007).miR-142-5p and miR-142-3p are both reported to play essential roles in cancer initiation and progression (Pahlavan et al. 2020). Chenfei Zhou et al. reported that miR-142-5p that derived from exosome could promote immune privilege by IDO in the tumor microenvironment (Zhou et al. 2021). The down-regulation of miR-142-5p could promote tumor metastasis by directly targeting CRY61 in gastric cancer (Yan et al. 2019). Besides, miR-142-3p induced apoptosis and suppressed breast cancer malignancy by targeting HMGA2 (Mansoori et al. 2021). In addition, miR-142-3p modulated cell migration and invasion via aerobic glycolysis mediated by PKM2 in colorectal cancer (Ren et al. 2021). In our previous study, miR-142-3p was identified as a tumor suppressor, which could inhibit breast cancer proliferation and led to G2/M arrest of cell cycle by regulation of CDC25C (Cao et al. 2016). However, the underlying mechanism is still unclear and needs further investigation.

Paired box protein 5 (PAX5) belongs to PAX gene family, which contains nine nuclear transcription factors and perform critical roles in various disease formation and cell development (Calderon et al. xxxx; Medvedovic et al. 2011; Yu et al. 2021). Wu X et al. demonstrated that PAX5 transcriptionally activated LncRNA FOXP4-AS1 and promoted the progression of prostate cancer by regulating miR-3184-5p (Wu et al. 2019). It has been revealed that the hypermethylation of PAX5 promoter region was commonly existed in multiple cancers and became the crucial factor of the occurrence and development of tumors (Haghverdi and Moslemi 2018). Liu X et al. reported that PAX5 served as a novel biomarker of breast cancer and the expression of PAX5 was silenced or reduced by methylation in breast cancer (Li et al. 2018a). Nevertheless, the specific regulation mechanism of PAX5 in breast cancer is still uncertain.

In this study, we investigated the mechanism of miR-142-5p/3p in breast cancer progression and identified the PAX5 as the upstream regulator of miR-142. Besides, the expression of PAX5 was regulated by methylation of its promoter region induced by DNMT1 and ZEB1, which were target genes of miR-142-5p and miR-142-3p, respectively. In summary, we exhibited a feedback regulation loop formed by PAX5, miR-142, and DNMT1/ZEB1, which play crucial roles in breast cancer development.

## Materials and methods

### Breast cancer samples

Breast cancer samples (n = 236 cases) and their paired adjacent normal tissues were collected from Tianjin Medical University Cancer Institute and Hospital from 2012 to 2022. The tissues were collected immediately after mastectomies and stored at − 80 °C for following analysis. The protocols were reviewed and approved by the Ethical Committee of Tianjin Medical University Cancer Institute and Hospital. All patients were informed and signed consent.

### Cells, plasmids, siRNAs and transfection

The normal breast epithelium cells ZR-75-1 and breast cancer cells MCF-7, T47D, BT549, MDA-231 and SK-BR-3 cells were purchased from ATCC (Manassas, VA). The cells were cultured in corresponding mediums with 1% penicillin/streptomycin (Gibco, USA) and 10% fetal bovine serum (FBS) (Gibco, USA) in a 37 °C incubator at the 5% CO_2_ atmosphere.

The expression plasmids of PAX5, DNMT1 and ZEB1 were purchased from Genechem (Shanghai, China). The miR-142-5p/3p mimic and inhibitor, small interfering RNAs (siRNAs) targeting PAX5, DNMT1 and ZEB1 were obtained from RiboBio (Guangzhou, China). Transfection was performed using Lipofectamine 3000 (Invitrogen, USA) according to the manufacture’s recommendations.

### Immunoblotting (IB) and co-immunoprecipitation (co-IP)

The protein from cells were extracted by RIPA lysis buffer with 1% protease inhibitor cocktail. The protein concentrations were detected by using BCA kit (Thermo, USA). For western blot, 30 μg protein lysates were separated through 10% SDS-PAGE electrophoresis and transferred to PVDF membranes. The target proteins were immunoblotted by corresponding antibodies and visualized by ECL reagent (Millipore, Bedford, MA, USA). For co-immunoprecipitation (co-IP), the cell lysates were added with 40 μg dynabeads protein G (Life Technologies) as well as 3 μg specific antibodies and incubated overnight at 4 °C. The beads were washed 3 times by PBS and heating with equal volume lysis buffer. Then bound proteins and 10% inputs were detected by IB described before. The detail information of antibodies involved in this article was added in Additional file 1.

### RNA extraction and qRT-PCR

Total RNA was extracted from tissues or cells by using Trizol reagent (Invitrogen, USA) and estimated by NanoDrop 2000 spectrophotometer (Thermo, USA). For mRNA detection, The RNA was reversed by a First Strand cDNA Synthesis kit (TakaRa, China), and the quantitative Real-time PCR was conducted by using GoTaq qPCR Master Mix (Promega). For miRNA detection, the RNA was reversed by TaqMan MicroRNA Reverse Transcription kit, while the qRT-PCR was performed by using miRNA qRT-PCR Starter kit (RiboBio, China) according to the manufacturer’s protocols. The primers involved were listed in Additional file 1.

### Cell proliferation assay

For colony formation assay, we planted 800 cells into 6-well plates and incubated for approximately 2 weeks, until the colonies reached appropriate size. Then the cells were washed by PBS, fixed by 4% paraformaldehyde, and stained by 1% crystal violet.

For MTT assay, we seeded 3 × 10^3^ cells into 96-well plates with 200 μL normal medium. The cells were added with 20 μL MTT solution at the same time point of the 1–5 days. After incubation of another 4 h, the medium was abolished, and the precipitate was dissolved in DMSO. After 10 min of shaking, the absorbance at 570 nm wavelength was detected by a microplate reader (BioRed).

For EdU assay, we planted 7 × 10^4^ cells into 24-well plates overnight. When the cells are fully attached, the 100 μL EdU solution (RiboBio, China) was added at the concentration of 50 μM and incubate for 2 h. Then the cells were fixed and stained by Apollo fluorescent dye solution (RiboBio, China). Finally, the nuclear DNA was stained by Hoechst 33342 solution (RiboBio, China). The images were captured and analyzed by a fluorescence microscope (Zeiss).

### Flow cytometry

Breast cancer cells after 48 h of transfection were harvested and washed by PBS. 95% ethanol was utilized for cells fixation at 4 °C overnight. Then the cells were stained by 5 μL of propodeum iodide solution (Thermo) for 15 min avoid light. The Beckman Coulter EPICS flow cytometer (Krefeld, Germany) was used for on-board detection of cell cycle.

### DNA extraction and bisulfite sequencing PCR (BSP)

The DNA from cells were extracted via a genomic DNA isolation kit (Thermo Scientific, USA) according to the manufacturer’s instruction. The DNA conversion was induced by a EpiTect bisulfite kit (Qiagen). The bisulfite-converted sequences of PAX5 were amplified and resolved by QIAquick gel extraction kit (Qiagen) and cloned into a pGEM-T easy vector (Promega). Ten colonies from each ligation were randomly selected and sequenced. The methylation status was estimated by the present of T (non-methylated) or C (methylated). The primers used were supplemented in Additional file 1.

### Chromatin immunoprecipitation (ChIP) analysis

The cells were were fixed by 1% formaldehyde and treated with 0.125 M glycine. The Upstate Biotechnology kit was used for following process. For immunoprecipitation of protein/DNA complex, 1μL specific antibodies were added into each reaction system, while the non-specific antibody of IgG was set as a negative control. Then the samples were conducted with PCR amplification and the fold changes of % input was used for analysis. Related primers used for ChIP assay were listed in Additional file 1.

### Dual-luciferase reporter assay

The putative binding sites of miR-142 promoter, PAX5 promoter, as well as 3’UTRs of DNMT1 and ZEB1 were cloned into psiCHEK2 luciferase reporter plasmid and verified by sequencing. Mutations were generated by using the QuikChange Mutagenesis Kit (Stratagene) in accordance with the manufacturer’s suggestion. The primers used for PCR amplification and mutagenesis were given in Additional file 1. The luciferase reporter plasmids were co-transfected into 293FT cells and incubated for 48 h. The Dual-Luciferase Reporter Assay Kit (Transgene) were used to detect Firefly and Renilla luciferase activities by an Orion II luminometer (Berthold).

### miRNA pull-down assay

The biotinylated miR-142-5p and miR-142-3p, as well as their controls were purchased form RiboBio (Guangzhou, China). The 100 nM biotinylated miRNAs were transfected into breast cancer cells and incubated for 48 h. The magnetic beads were used for pulling down the complex of biotinylated miRNA, which was isolated by Trizol (Thermo). The enrichment of DNMT1 and ZEB1, as well as GAPDH were detected by qRT-PCR, which primers were given in Additional file 1.

### Immunohistochemistry (IHC)

The sections of human and mice were deparaffinized, dehydrated and undergone heat-induced antigen retrieval. After the block of endogenous peroxidase activity by 3% H_2_O_2_, the sections were treated with 5% BSA at room temperature for 1 h. The primary antibodies were used for incubating sections at 4 °C overnight. The next day, the sections were washed by PBS, and incubated with corresponding secondary antibodies. After visualized by 3,3′-diaminobenzidine (DAB) reagent, the sections were stained with hematoxylin and differentiated by hydrochloric acid alcohol. Finally, all sections were covered by neutral gum and captured by a light microscope (Olympus).

### Xenograft

In vivo experiments were performed using 5-week-old female SCID mice purchased from SPF Biotechnology (Beijing, China). The breast cancer cells were injected into the mammary fat pads at the number of 5 × 10^6^ cells per individual in 150μL PBS, and each single group contained six mice. The tumor growth was detected and recorded once a week, until the tumor volumes reached appropriate sizes (nearly 35 days). Then the mice were executed, and the tumors were stripped and embedded in paraffin. The sections of mice tumor were conducted with hematoxylin–eosin (H&E) staining and immunohistochemical (IHC) analysis. The animal experiments were reviewed and approved by the Ethics Committee of Tianjin Medical University Cancer Institute and Hospital.

### Statistical analysis

All data was shown as mean ± standard deviation (SD) and analyzed by Prism 8 (Graph pad Software, Inc., La Jolla, CA). Student’s t-test was used for statistical analysis. p < 0.05 was considered with statistical significance.

## Results

### miR-142-5p/3p was a direct target of PAX5

**Fig. 1:**
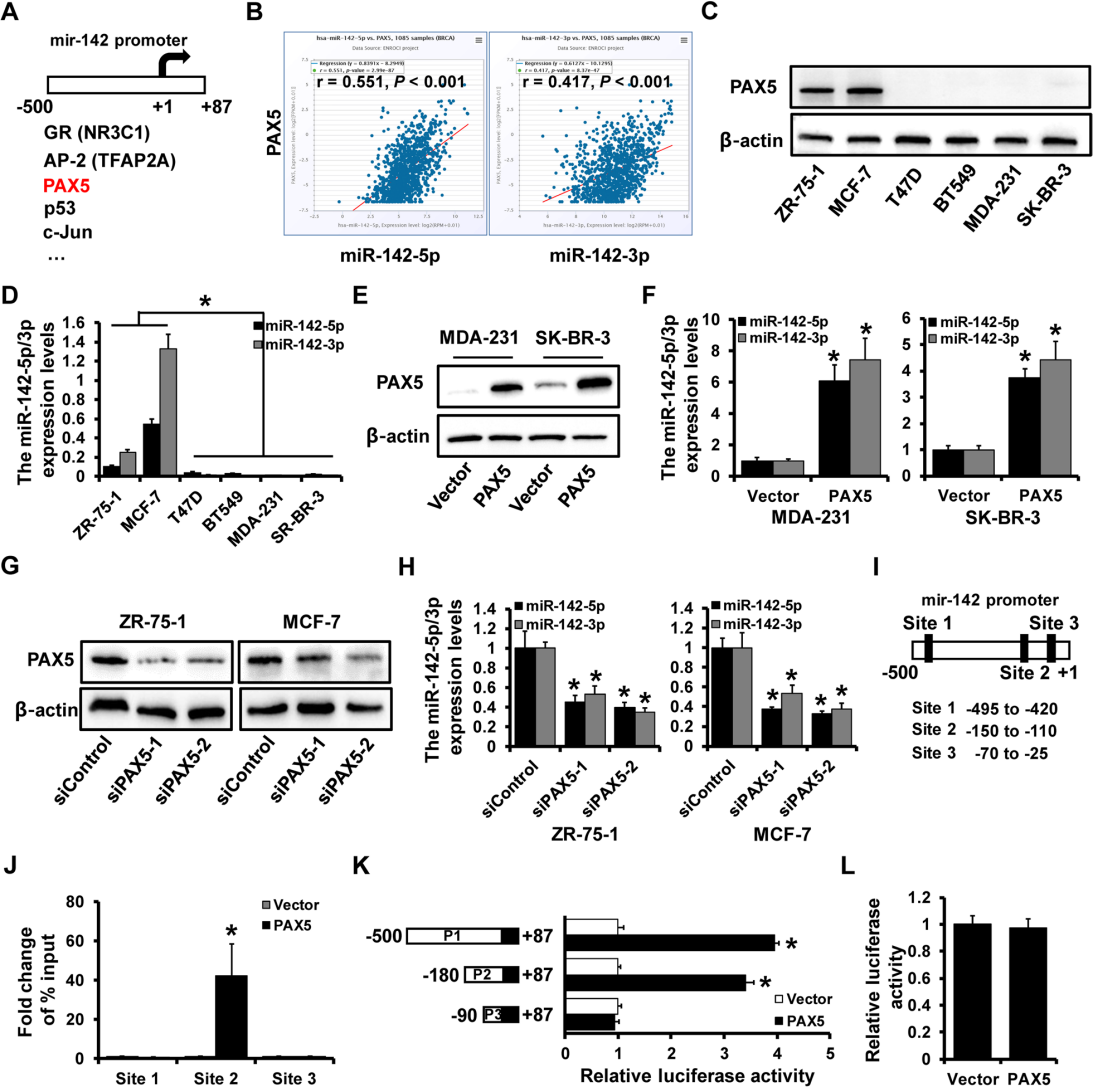
miR-142-5p/3p was a direct target of PAX5. **A** The candidates of miR-142 upstream regulator by ALGGEN. **B** The correlation between PAX5 with miR-142-5p and miR-142-3p, respectively. **C** The expression level of PAX5 in breast cancer cell lines detected by western blot. **D** The expression levels of miR-142-5p/3p in breast cancer cells detected by qRT-PCR. **E** The construction of PAX5-overexpressed MDA-231 and SK-BR-3 cells verified by western blot. **F** The expression levels of miR-142-5p/3p in PAX5-overexpressed MDA-231 and SK-BR-3 cells detected by qRT-PCR. **G** The construction of PAX5-delepted ZR-75-1 and MCF-7 cells verified by western blot. **H** The expression levels of miR-142-5p/3p in PAX5-delepted ZR-75-1 and MCF-7 cells detected by qRT-PCR. **I** The putative binding sites of PAX5 on miR-142 promoter region. **J** The interaction of putative binding sites of miR-142 promoter with PAX5 analyzed by ChIP assay. **K** Three truncated length of putative binding sites (P1, − 500 to + 87; P2, − 180 to + 87; P3, − 90 to + 87) from miR-142 promoter were constructed. **L** The luciferase activity of PAX5 on mutated binding site of miR-142 promoter. *p < 0.05

In our previous study, miR-142-5p was identified as a tumor suppressor functioned by regulation of CDC25C. In order to find out the further mechanism and upstream regulator of miR-142, ALGGEN (http://alggen.lsi.upc.es/) was used and screened several potential genes (Fig. 1A). Then starBase v.3.0 was used to analyze the correlation between each candidate and miR-142-5p/3p, which results showed PAX5 presented the strongest positive correlation with both miR-142-5p and miR-142-3p (Fig. 1B), while other candidates without much significance (Additional file 1: Figure S1). For better acknowledgement of PAX5 expression pattern in breast cancer cell lines, western blot was performed and indicated that PAX5 was over-expressed in normal breast cell line ZR-75-1 and luminal A subtype breast cancer cell line MCF-7, while rarely expressed in other breast cancer cell lines (Fig. 1C). Consistent results were observed when detecting the miR-142-5p/3p expression pattern in breast cell lines by qRT-PCR (Fig. 1D). To investigate the expression correlation of PAX5 and miR-142, we transfected PAX5 expression plasmid into MDA-231 cells and SK-BR-3 cells and verified by western blot (Fig. 1E). qRT-PCR results showed that both miR-142-5p and miR-142-3p were up-regulated in PAX5 over-expression cell lines (Fig. 1F). Then, we transfected siRNAs that targeting PAX5 to construct PAX5-deleption ZR-75-1 cells and MCF-7 cells (Fig. 1G). It was observed that PAX5-deleption induced decrease of miR-142-5p/3p by qRT-PCR (Fig. 1H). Furthermore, three potential binding sites of PAX5 on miR-142 promoter region (− 500 to + 1) were predicted (Fig. 1I), Among which, the occupancy by PAX5 was detected in the site 2 (− 150 to − 110) region through ChIP analysis (Fig. 1J). To further investigate the regulation of PAX5 on miR-142, three truncated length of putative binding sites (P1, − 500 to + 87; P2, − 180 to + 87; P3, − 90 to + 87) from miR-142 promoter were constructed and co-transfected with PAX5 or vector into MCF-7 cells, which indicated that PAX5 was mainly affected by the luciferase activity of P2 region (Fig. 1K). Then, we mutated this predicted binding site of PAX5 and miR-142, the reported result of luciferase showed that PAX5 failed to increase the luciferase activity of mutant group (Fig. 1L). These findings indicated that both miR-142-5p and miR-142-3p were direct targets of PAX5.

### PAX5 inhibited breast cancer proliferation by targeting miR-142-5p/3p

**Fig. 2:**
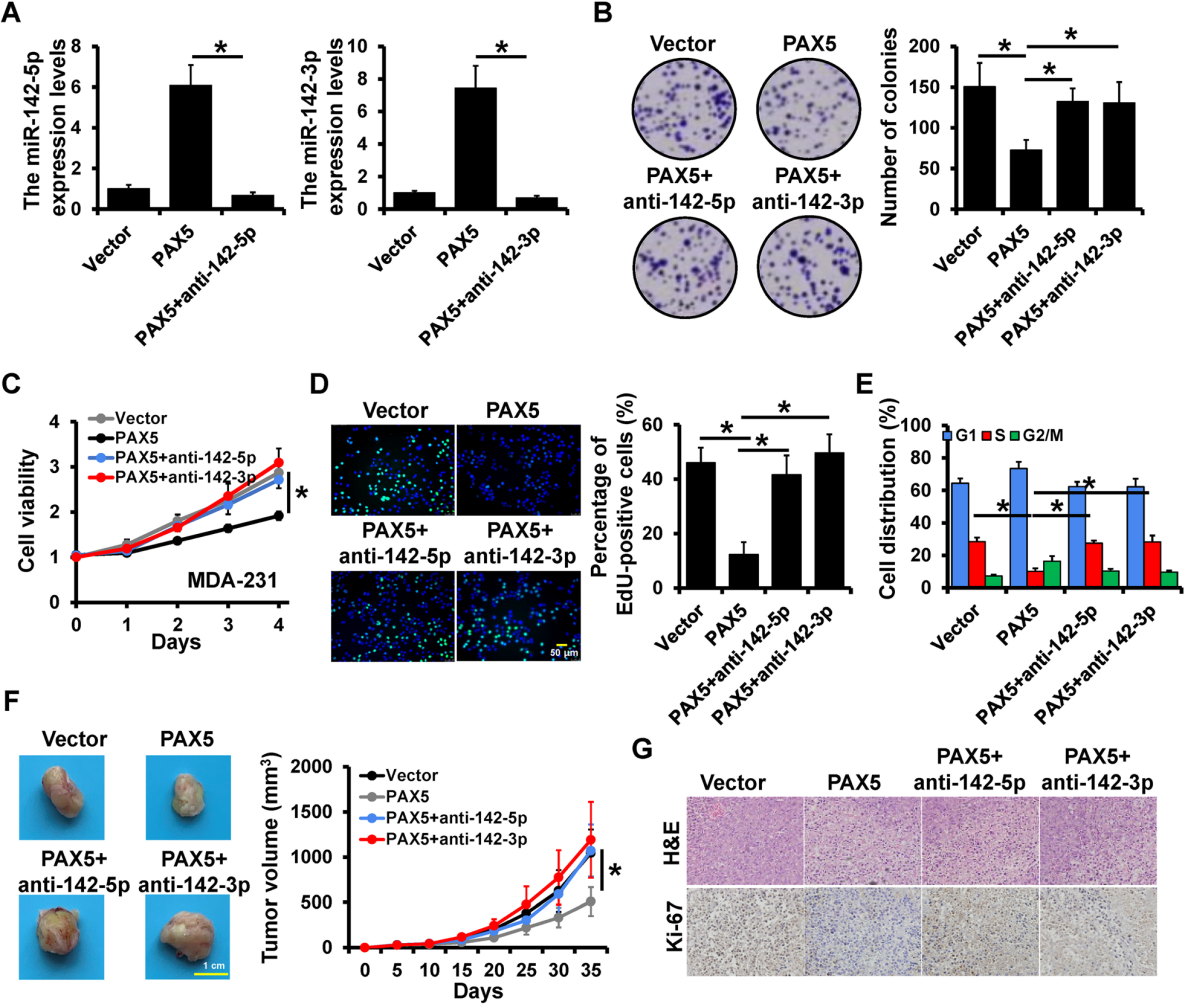
PAX5 inhibited breast cancer proliferation by targeting miR-142-5p/3p. **A** The construction of PAX5-overexpressed miR-142-5p-delepted (left) and PAX5-overexpressed miR-142-3p-delepted (right) MDA-231 cells verified by qRT-PCR. The cell proliferation assays were conducted, including colony formation assay (**B**), MTT assay (**C**) and EdU assay (**D**). **E** The distribution of cell cycle was detected by flow cytometry. **F** Representative photos and growth curve of tumors. **G** The H&E staining and Ki-67 expression levels of xenograft tumors detected by IHC. *p < 0.05

To investigate the effect and regulation of PAX5 in breast cancer progression, the PAX5 expression plasmid was transfected into MDA-231 cells with or without anti-142-5p/3p, and the expression levels of miR-142-5p/3p were detected by qRT-PCR (Fig. 2A). In colony formation assay, it was observed that over-expressed PAX5 significantly decreased the numbers of colonies, while the deletion of miR-142-5p and miR-142-3p could increase the number of colonies (Fig. 2B). The consistent results were also observed in MTT assay (Fig. 2C) and EdU assay (Fig. 2D) that the decreased cell viability and percentage of EdU positive cells induced by PAX5 over-expression were reversed by miR-142-5p/3p deletion. In the analysis of cell cycle, it was showed that PAX5 decreased the proportion of S phase while miR-142-5p/3p-depeltion reversed the proportion of S phase (Fig. 2E). In vivo, the PAX5-overexpressed and miR-142-delepted cells were subcutaneous injected into SCID mice, and it was observed that the tumor volumes were notably decreased by PAX5 over-expression and increased by miR-142-5p/3p-deletion (Fig. 2F). Then the tumors of mice were sectioned and stained with H&E and Ki-67 antibody by IHC, which results showed that PAX5 over-expression led to a down-regulation of Ki-67 expression, and the depletion of miR-142-5p/3p resulted in up-regulations of Ki-67 (Fig. 2G). In addition, we co-transfected siPAX5 and miR-142-5p/3p mimic into MCF-7 cells to further verify the regulation of PAX5 on miR-142 and observed consistent results (Additional file 1: Figure S2). These results indicated that PAX5 served as a tumor suppressor and functioned through the regulation of miR-142-5p/3p.

### PAX5 was hypermethylated by DNMT1

**Fig. 3:**
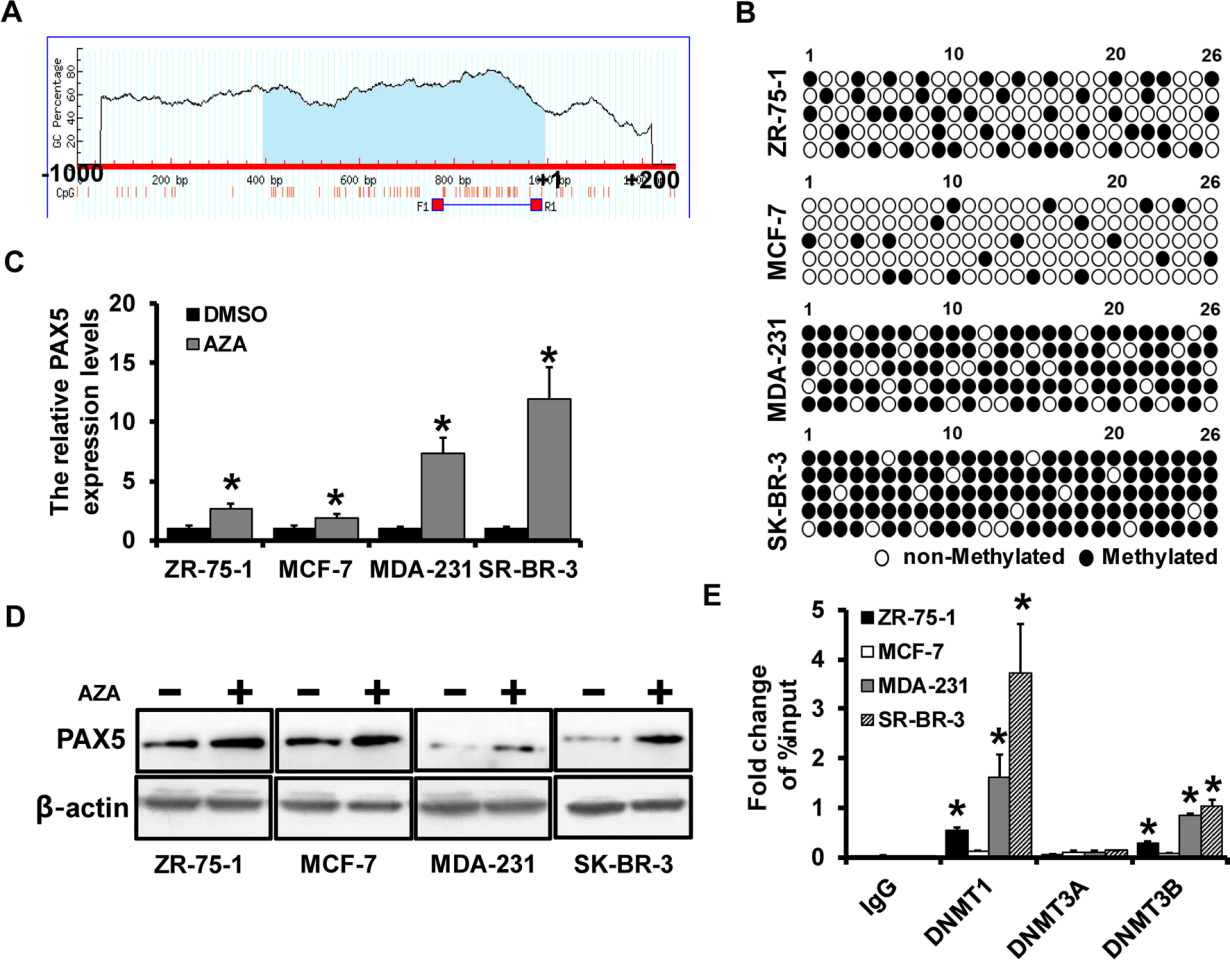
PAX5 was hypermethylated by DNMT1. **A** The existence of CpG island on PAX5 promoter region predicted by Meth primer. **B** The methylation status of breast cancer cells detected by BSP sequencing. The expression level of PAX5 after the treatment of AZA in breast cancer cell lines by qRT-PCR (**C**), and western blot (**D**). The interaction of DNMT1 and PAX5 detected by ChIP analysis. *p < 0.05

Next, we further investigated the regulation of PAX5 expression in breast cancer. The existence of CpG island was predicted on the promoter region of PAX5 by Meth primer (Fig. 3A). The BSP sequencing was conducted in four types of breast cell lines to detect the methylation status, which showed that PAX5 was hypomethylated in ZR-75–1 and MCF-7 cells, while hypermethylated in MDA-231 and SK-BR-3 cells (Fig. 3B). To confirm this, the methylated transferase inhibitor regent AZA was used to treat cells, and the expression of PAX5 was found to up-regulated after the treatment of AZA detected by qRT-PCR (Fig. 3C) and western blot (Fig. 3D). To find out the specific methylated transferase inhibitors that regulated PAX5, ChIP analysis was performed and showed that PAX5 significantly interacted with DNMT1 (Fig. 3E). In summary, the expression of PAX5 was regulated by the methylation of its promoter region induced by DNMT1.

### DNMT1 regulated PAX5 expression by ZEB1

**Fig. 4:**
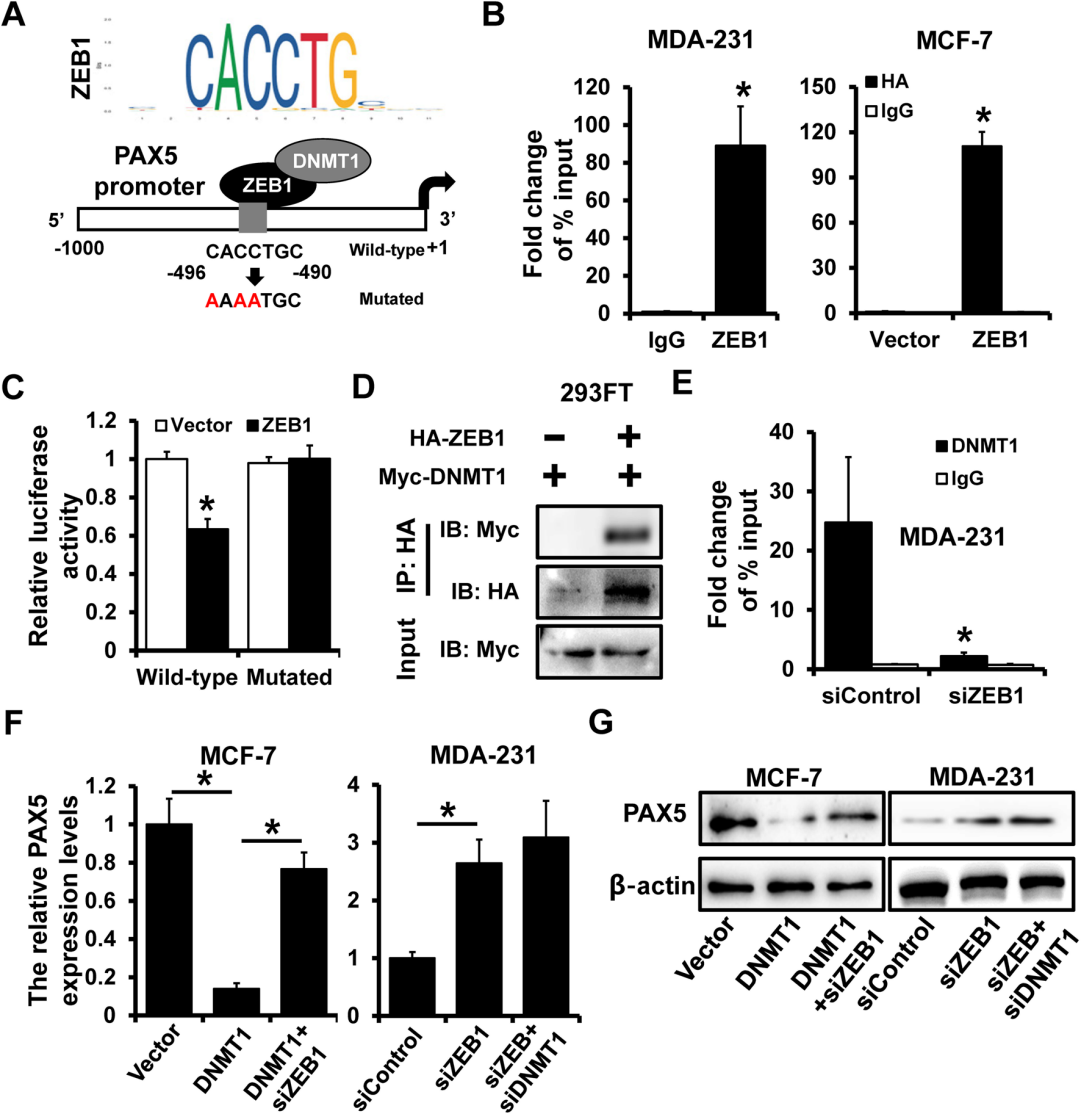
DNMT1 regulated PAX5 expression by ZEB1. **A** The putative binding of ZEB1 on PAX5 promoter region. **B** ChIP analysis of ZEB1 and PAX5 promoter in MDA-231 and MCF-7 cells. The interaction of ZEB1 and PAX5 promoter region detected by luciferase reporter assay (**C**), and co-IP analysis (**D**). **E** The fold change of DNMT1 on PAX5 promoter in ZEB1-delepted MDA-231 cells. The PAX5 expression level in DNMT1-overexpressed ZEB1-delepted MCF-7 cells (left), and in DNMT1-delepted ZEB1-delepted MDA-231 cells, detected by qRT-PCR (**F**) and western blot (**G**). *p < 0.05

The putative binding site of ZEB1 and PAX5 promoter was predicted by JASPAR (https://jaspar.genereg.net/) (Fig. 4A). The occupancy of ZEB1 on PAX5 promoter region was detected by ChIP analysis (Fig. 4B), including endogenous interaction in MDA-231 cells (left) and exogenous interaction in MCF-7 cells (right). To further clarify the regulation of ZEB1 on PAX5, we cloned wild-type and mutated binding region of ZEB1 and PAX5 promoter into psiCHEK2 luciferase reporter plasmid. The over-expression of ZEB1 led to a nearly 40% decrease of luciferase activity of wild-type group, while no significance in mutated group (Fig. 4C). Besides, to investigate the correlation between ZEB1 and DNMT1, the HA-specific co-immunoprecipitation assay was performed in 293FT cells and showed that ZEB1 could interact with DNMT1 (Fig. 4D). DNMT1 was able to bind to the promoter region of PAX5 as described before, however, after the transfection of siZEB1, the binding of DNMT1 and PAX5 promoter was remarkably reduced by ChIP assay (Fig. 4E). The mechanism behind deserved further exploration. We co-transfected DNMT1 and siZEB1 as well as their corresponding controls into MCF-7 cells, which result showed that DNMT1 significantly decreased the mRNA expression level of PAX5, but DNMT1 over-expression with ZEB1-depletion enhanced the mRNA expression level of PAX5 (Fig. 4F, left). At the same time, we co-transfected siZEB1 with siDNMT1 into MDA-231 cells and observed that siZEB1 increased the expression level pf PAX5, while siZEB1 with siDNMT1 made no specific change on PAX5 expression (Fig. 4F, right). The consistent results were obtained by western blot (Fig. 4G), which indicated that ZEB1 was the middle regulator between DNMT1 and PAX5. Taken together, these findings revealed that DNMT1 inhibited the expression of PAX5 by regulation of its upstream transcriptional factor ZEB1.

### The existence of PAX5-miR-142-DNMT1/ZEB1 feedback loop

**Fig. 5:**
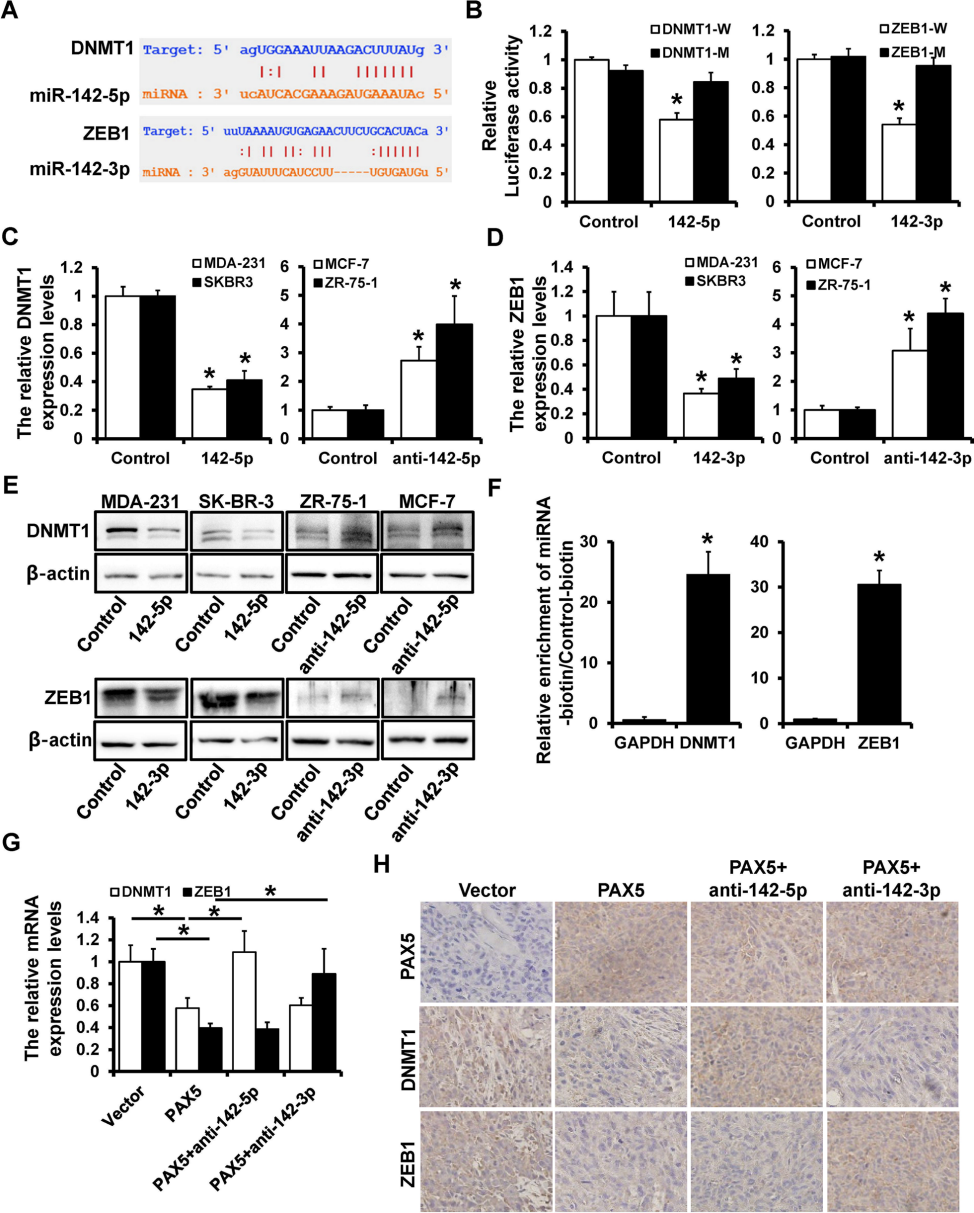
The existence of PAX5-miR-142-DNMT1/ZEB1 feedback loop. **A** The predicted binding sites of miR-142-5p on 3’UTR of DNMT1 and miR-142-3p on 3′UTR of ZEB1 by TargetScan. **B** The regulation of miR-142 on DNMT1 and ZEB1 detected by luciferase reporter assay. **C** The expression level of DNMT1 in miR-142-5p-overexpressed MDA-231 and SK-BR-3 cells (left), and in miR-142-5p-delepted MCF-7 and ZR-75-1 cells (right). **D** The expression level of ZEB1 in miR-142-3p-overexpressed MDA-231 and SK-BR-3 cells (left), and in miR-142-3p-delepted MCF-7 and ZR-75-1 cells (right) detected by qRT-PCR and western blot (**F**). **E** Pull-down assay by biotined-miR-142-5p/3p. **G** The relative mRNA expression levels of DNMT1 and ZEB1 in PAX-overexpressed, PAX5-overexpressed with miR-142-5p-delpeted, and PAX5-overexpressed with miR-142-3p-delpeted cells by qRT-PCR. **H** The expression levels of DNMT1 and ZEB1 in xenograft tumors. *p < 0.05

The putative binding sites of miR-142-5p on DNMT1 and miR-142-3p on ZEB1 were predicted by Targetscan (Fig. 5A). The wild-type and mutant binding site of DNMT1 or ZEB1 were co-transfected with miR-142-5p or miR-142-3p, respectively. The luciferase reporter assay indicated that miR-142-5p/3p reduced the luciferase activity of wild-type DNMT1 and ZEB1 (Fig. 5B). For further investigation, miR-142-5p was over-expressed in MDA-231 and SKBR3 cells, which down-regulated the expression level of DNMT1 (Fig. 5C, left); The anti-miR-142-5p was transfected into MCF-7 and ZR-75-1 cells and led to an up-regulation of DNMT1 (Fig. 5C, right). Similarly, the miR-142-3p mimic and inhibitor were transfected into four types of breast cancer cells, respectively. The over-expression of miR-142-3p increased the expression of ZEB1, while miR-142-3p-depletion reduced the expression of ZEB1 by qRT-PCR (Fig. 5D). Consistent results were observed by western blot (Fig. 5F). The pull-down assay indicated higher DNMT1 enrichment ratio by biotin-miR-142-5p and higher ZEB1 enrichment ratio of ZEB1 by biotin-miR-142-3p (Fig. 5E). To find out the expression regulation of PAX5 and DNMT1 or ZEB1, we constructed PAX5 over-expressed, PAX5-over-expressed and miR-142-5p-delepted, and PAX5-over-expressed and miR-142-3p-delepted cells, which results showed that the over-expression of PAX5 could both down-regulated DNMT1 and ZEB1 expression, while the DNMT1 expression was increased in cells co-transfected with anti-miR-142-5p, ZEB1 expression was increased in cells co-transfected with anti-miR-142-3p (Fig. 5G). For further clarification, IHC was performed and indicated that PAX5 over-expression led to decrease expression of DNMT1 and ZEB1, while their staining intensity significantly enhanced in miR-142-5p-delepted or miR-142-3p-delepted tissues, respectively (Fig. 5H). These findings revealed that DNMT1 was the direct target of miR-142-5p, while ZEB1 was the direct target of miR-142-3p. PAX5 could regulate the expression of DNMT1 and ZEB1 by regulation of miR-142. Taken together, PAX5-miR-142-DNMT1/ZEB1 formed a feedback loop in regulation of breast cancer progression.

### Clinical relevance

**Fig. 6:**
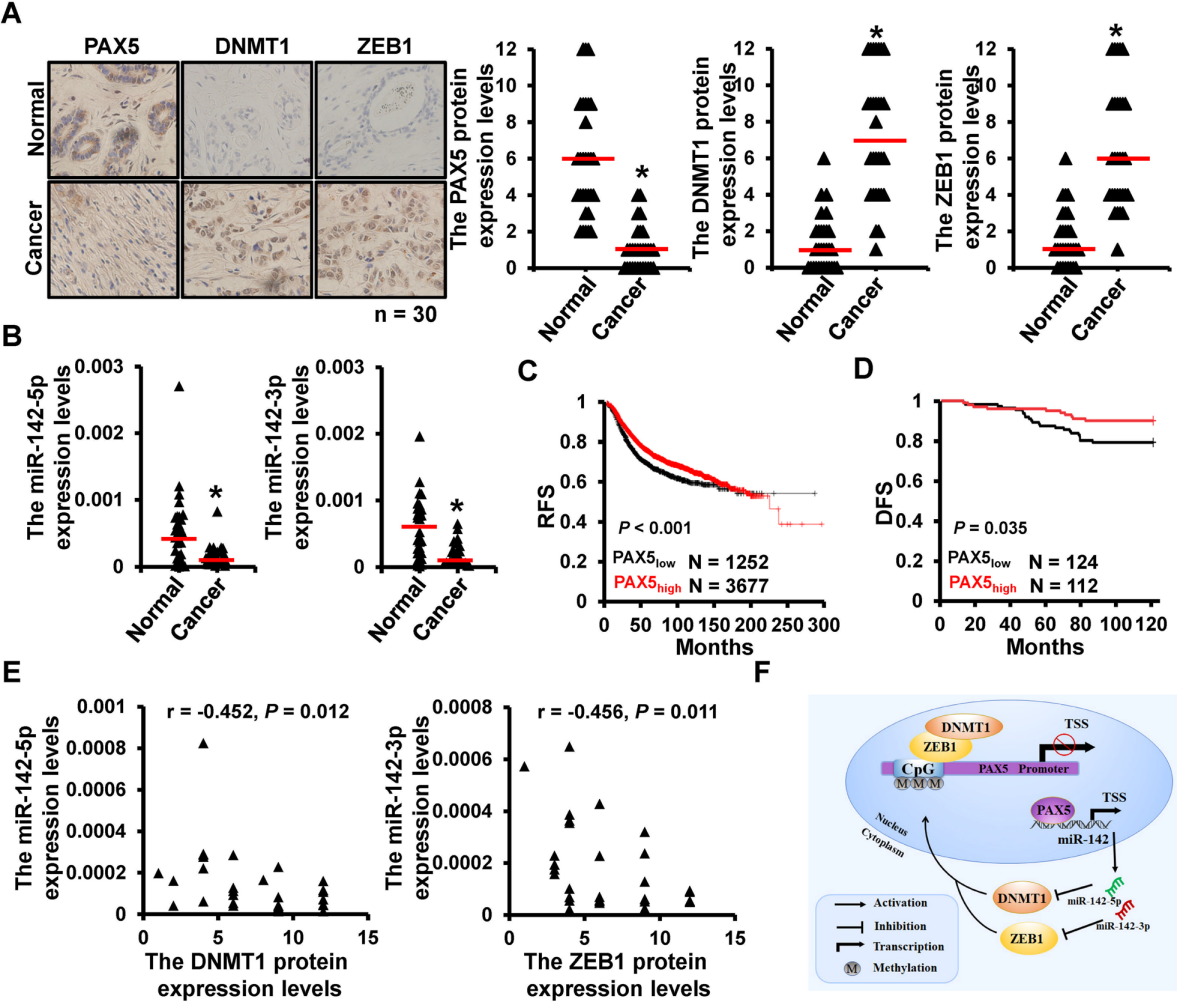
Clinical relevance. **A** The expression levels of DNMT1 and ZEB1 in breast cancer tissues compared with adjacent normal tissues. **B** The expression levels of miR-142-5p (left) and miR-142-3p (right) in breast cancer tisssues. **C** The correlation of recurrence-free survival (RFS) and PAX5 expression predicted by KMplotter. **D** The correlation of disease-free survival (DFS) and PAX5 expression measured by 236 cases clinical profiles. **E** The expression correlation between miR-142-5p/3p and DNMT1/ZEB1. *p < 0.05

To investigate the expression pattern of PAX5, DNMT1 and ZEB1, as well as miR-142, 30 cases of breast cancer tissues and their paired adjacent normal tissues were collected and stained by IHC. The expression of PAX5 was significantly down-regulated in breast cancer tissues, while the expression levels of DNMT1 and ZEB1 were both up-regulated in breast cancer tissues, compared with normal tissues (Fig. 6A). Meanwhile, the expression levels of miR-142-5p/3p were detected by qRT-PCR, which showed a notably decrease in breast cancer tissues (Fig. 6B). Furthermore, the KMplotter was utilized for predicting the prognostic correlation of PAX5, which indicated that higher expression of PAX5 was related to worse clinical outcome (Fig. 6C). Besides, 236 cases of breast cancer patients (including 124 cases with PAX5 low expression and 112 cases with PAX5 high expression) were collected and analyzed, which results were consistent with the prediction that PAX5 over-expression was associated with worse prognosis (Fig. 6D). In addition, the expression correlations of miR-142 with DNMT1 or ZEB1 were analyzed, which results indicated that miR-142-5p was negatively correlated with DNMT1, while miR-142-3p was negatively associated with ZEB1 (Fig. 6E). These results revealed the expression pattern of PAX5, DNMT1, ZEB1 and miR-142 in breast cancer tissues, and indicated the regulation of the feedback loop.

## Discussion

miR-142-5p and miR-142-3p were reported to play crucial roles in various cancer progressions (Soares et al. 2017; Berrout et al. 2017; Jin et al. 2023; Huang et al. 2023). For instance, miR-142-5p was up-regulated by LSD1-deleption and repressed cell migration via targeting CD9 in gastric cancer (Zhao et al. 2020). In addition, miR-142-3p served as a tumor suppressor by regulation of RAC1/PAK1 signaling in breast cancer (Xu et al. 2020). Besides, a latest study demonstrated that miR-142-3p was the target of ST8SIA6-AS1 and involved in hepatocellular carcinoma progression (Feng et al. 2023). In our previous study, we demonstrated the inhibition of miR-142-3p on breast cancer cell cycle by targeting CDC25C (Cao et al. 2016). However, its further functional mechanism was still unclear. Herein, in this study, we further investigated the underlying mechanism of miR-142-3p and its another mature miR-142-5p in breast cancer progression.

In this study, PAX5 was identified as the upstream regulator of miR-142-5p/3p by ALGGEN, which was positively correlated with miR-142-5p/3p. PAX5 is a nuclear transcription factor that involved in multiple biology process and cancer progression including breast cancer (Li et al. 2023). For example, Benzina et al. reported that PAX5 was able to regulate breast cancer malignant processes through the disruption of FAK signaling (Benzina et al. 2016). In current study, we demonstrated that PAX5 could binding with the promoter of miR-142 at the region from − 150 to − 110 detected by ChIP analysis and luciferase reporter assay. Furthermore, PAX5 functioned as a tumor suppressor by directly regulating miR-142-5p/3p by experiments both in vitro and in vivo.

Next, we further explored the regulator of PAX5 in breast cancer. Previous studies have revealed potential mechanism of the regulation of PAX5 expression. As reported by Li et al., PAX5 was frequently down-regulated by methylation of its promoter region in breast cancer (Li et al. 2018a). Besides, a significantly higher methylation of PAX5 was also detected in hepatocellular carcinoma (HCC) (Mzik et al. 2016). Thus, we predicted the existence of CpG island of PAX5 promoter by Meth primer, and detected the methylation status of PAX5 in four subtypes of breast cells by BSP sequencing, which results showed the abnormal methylation of PAX5 in breast cancer. Furthermore, we investigated the interaction of PAX5 with three common methyltransferases, including DNMT1, DNMT3A and DNMT3B by ChIP analysis, which indicated that PAX5 was heavily combined with DNMT1 in MDA-231 and SK-BR-3 cells.

For further investigation, we attempted to find out the specific regulator that led to the methylation of PAX5 promoter region. Ectopic zinc-finger E-box binding homeobox 1 (ZEB1) was commonly reported as a transcription factor that involved in DNA methylation (Kitz et al. 2021). Yiying Chen et al. demonstrated that ZEB1 induced hypermethylation of Ddr1 by interaction with DNMT3B (Chen et al. 2021). Besides, ZEB1 was also reported to repress the expression of ER-α transcriptionally by forming a ZEB1/DNMT3B/ HDAC1 complex on the ER-α promoter, which led to its hypermethylation in breast cancer(Zhang et al. 2017). Thus, we predicted the biding site of ZEB1 and PAX5 promoter region by JASPAR and verified through ChIP assay and luciferase reporter assay. Furthermore, ZEB1 could also interacted with DNMT1, which was detected by co-IP. To clarify the correlation of ZEB1 and DNMT1, ChIP analysis was performed and showed a decrease fold change of DNMT1 on PAX5 promoter region in ZEB1-delepted cells. Besides, qRT-PCR and western bolt were performed, which results indicated that DNMT1 regulated PAX5 expression by targeting ZEB1.

Finally, we investigated the down-stream targets of miR-142-5p/3p. Interestingly, it has been reported that miR-142-3p could affect progression of breast cancer by directly targeting ZEB1 (Li et al. 2018b). Consistently, miR-142-3p was also reported to influence the epithelial-mesenchymal transition by targeting ZEB1 in hepatocellular carcinoma (HCC) (He et al. 2018). While miR-142-5p was found to inhibit the cell invasion and migration by targeting DNMT1 in breast cancer (Li et al. 2022). These studies were encouraging. Thus, we focused on the regulation of miR-142-5p on DNMT1 and the regulation of miR-142-3p on ZEB1, which were verified by luciferase reporter assay, qRT-PCR, pull-down assay by biotin-miRNA, western blot, as well as mice specimen.

Taken together, we revealed a feedback loop of PAX5 and miR-142, which could affect breast cancer progression by regulating DNMT1 and ZEB1 (Fig. 6). Besides, these findings were further verified in clinical specimen.

## Conclusion

In summary, we demonstrate the existence of PAX5-miR-142-5p/3p-DNMT1/ZEB1 feedback loop in regulation of breast cancer. These findings might bring novel biomarkers and provide emerging strategies for breast cancer therapy.

## Supplementary Information

**Additional file 1: Figure S1. **The correlation between other candidates with miR-142-5p/3p, including AP-2, p53, GRand c-Junpredicted by starBase v.3.0. **Figure S2.**The construction of PAX5-delepted with miR-142-5p/3p-overexpressed cells verified by qRT-PCR. The cell proliferation assays were performed, including colony formation assay, MTTand EdU assay. The distribution of cell cycle was analyzed by flow cytometry. *p < 0.05. **Table S1.** Antibodies used for study. **Table S2.** Oligonucleotides of miRNAs and siRNAs. **Table S3. **Oligonucleotides used for RT-qPCR. Table S4. Oligonucleotides used for ChIP and methylation specific PCR.

## Data Availability

All data generated or analyzed during this study are included in this published article and its supplementary information files.
