# Supplementary material for: PAX5-miR-142 feedback loop promotes breast cancer proliferation by regulating DNMT1 and ZEB1
Source: Mol Med. 2023 Jul 4;29:89. doi: 10.1186/s10020-023-00681-y (PMC10320939; doi:10.1186/s10020-023-00681-y)
Supplement: Supplementary file 1 — Additional file 1: Figure S1. The correlation between other candidates with miR-142-5p/3p, including AP-2, p53, GRand c-Junpredicted by starBase v.3.0. Figure S2.The construction of PAX5-delepted with miR-142-5p/3p-overexpressed cells verified by qRT-PCR. The cell proliferation assays were performed, including colony formation assay, MTTand EdU assay. The distribution of cell cycle was analyzed by flow cytometry. *p < 0.05. Table S1. Antibodies used for study. Table S2. Oligonucleotides of miRNAs and siRNAs. Table S3. Oligonucleotides used for RT-qPCR. Table S4. Oligonucleotides used for ChIP and methylation specific PCR. [file 10020_2023_681_MOESM1_ESM.pdf]

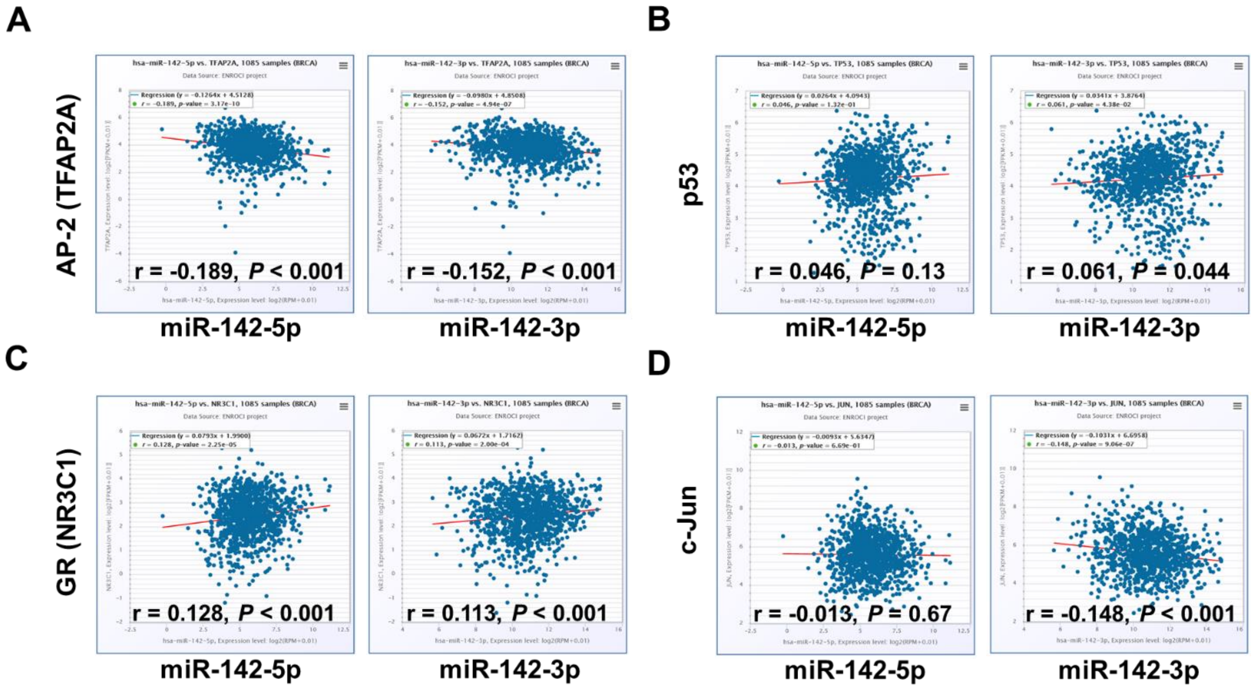

Figure S1. The correlation between other candidates and miR-142-5p/3p, including AP-2 (A), p53 (B), GR (C) and c-Jun (D) predicted by starBase v.3.0.

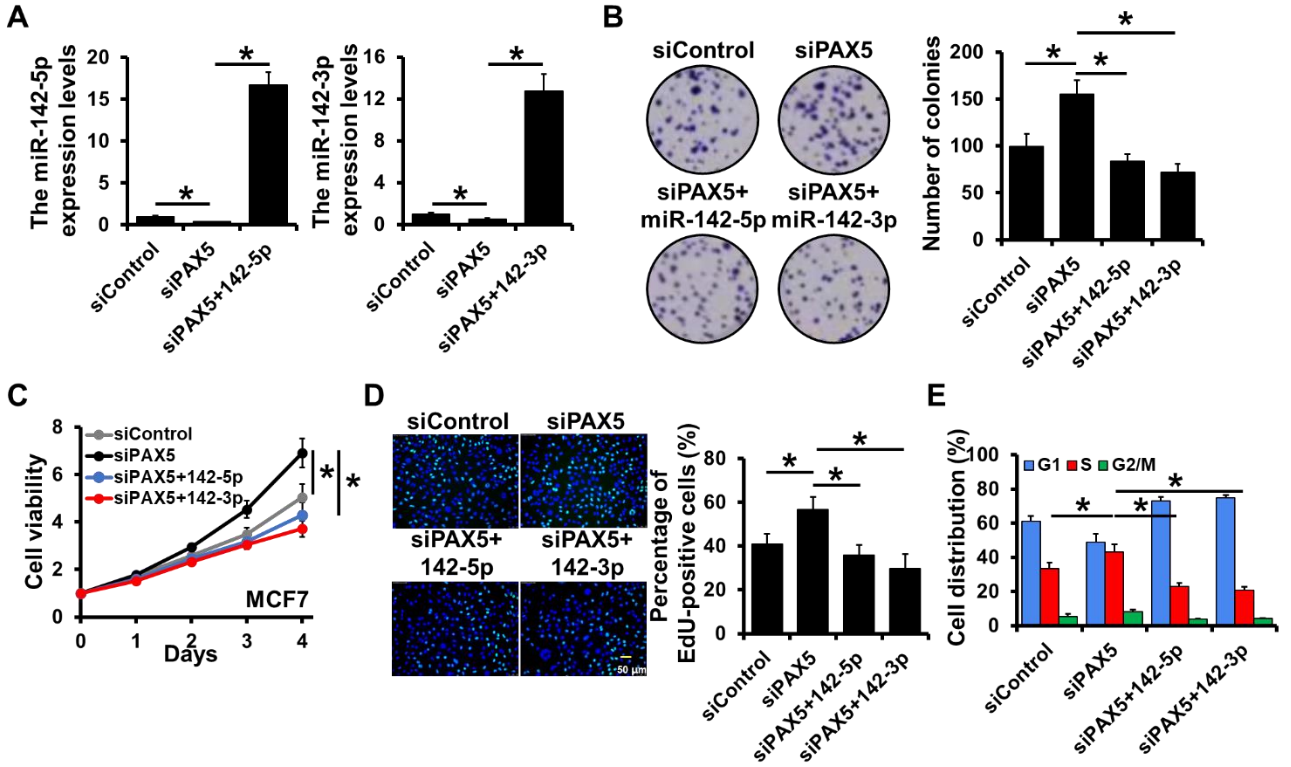

Figure S2. (A) The construction of PAX5-depleted with miR-142-5p/3p-overexpressed cells verified by qRT-PCR. The cell proliferation assays were performed, including colony formation assay (B), MTT (C) and EdU assay (D). The distribution of the cell cycle was analyzed by flow cytometry. *P < 0.05.

Table S1. Antibodies used for study

Name	Source	Catalog
HA	Immunoway	YM3003
Myc	Immunoway	YM3203
β-Actin	Immunoway	YM3028
DNMT1 (D63A6) XP® Rabbit mAb	Cell Signaling	5032
DNMT3A (D2H4B) Rabbit mAb	Cell Signaling	32578
DNMT3B (E8A8A) XP® Rabbit mAb	Cell Signaling	57868
PAX5	Abcam	ab227635
Anti-ZEB1 antibody	Abcam	ab180905

Table S2. Oligonucleotides of miRNAs and siRNAs

mimic control	UGUACCAAUUUCCAGUGGAGAU
inhibitor control	AUGGUGUUAUCAAGUGUAACAG
miR-142-5p mimics	CAUAAAGUAGAAAGCACUACU
miR-142-5p inhibitor	AGUAGUGCUUUCUACUUUAUG
miR-142-3p mimics	UGUAGUGUUUCCUACUUUAUGGA
miR-142-3p inhibitor	UCCAUAAAGUAGGAAACACUACA
siControl	UUCUCCGAACGUGUCACGU
siZEB1	GCUGUUGUUCUGCCAACAGUU
siDNMT1	GGAGAACGGUGCUCAUGCUU
siPAX5-1	AAUCGCUGAAUAUAAACGCCA
siPAX5-2	AACCAGUCCCAGCUUCCAGUC

Table S3. Oligonucleotides used for RT-qPCR

Name	Sequence (5' to 3')
miR-142-5p	CATAAAGTAGAAAGCACTAC<br>GAACATGTCTGCGTATCTC
miR-142-3p	TGCGGTGTAGTGTTTCCTACTT<br>CCAGTGCAGGGTCCGAGGT
U6	CTCGCTTCGGCAGCACA<br>AACGCTTCACGAATTTGCGT
PAX5 up<br>PAX5 low	TGGCAGGTATTATGAGACAGG<br>CAGGCAAACATGGTGGGATT
DNMT1 up	CAACGAGTCTGGCTTTGAGA

DNMT1 low	GACACAGGTGACCGTGCTTA
ZEB1 up<br>ZEB1 low	TCAAAAGGAAGTCAATGGACAA<br>GTGCAGGAGGGACCTCTTTA
GAPDH up<br>GAPDH low	CAAGGTCATCCATGACAACTTTG<br>GTCCACCACCCTGTTGCTGTAG

Table S4. Oligonucleotides used for ChIP and methylation specific PCR

Name	Sequence (5' to 3')
PAX5 site 1 up<br>PAX5 site 1 low	GCTGGGGACTTAGGCCCTGG<br>ACCTTCAGTTCTGTGAACCC
PAX5 site 2 up<br>PAX5 site 2 low	GAAGAGGGAACTGAAGAGGA<br>CTTGTGGCTTCCTAAGATCC
PAX5 site 3 up<br>PAX5 site 3 low	GGATCTTAGGAAGCCACAAG<br>CCTCCAGTGCTGTTAGTAGT
ZEB1 site up<br>ZEB1 site low	GCAATAGTCAGGACCCCAAC<br>TGACATCTCCATGTGCAAAC
BSP up<br>BSP low	TTTTAATTTTTATAAAAGTTTGGGG<br>CCATATAAAATCATATCCTATTCTC
